# New Genetic Variants of *Leptospira* spp Characterized by MLST from Peruvian Isolates

**DOI:** 10.1155/2022/4184326

**Published:** 2022-09-22

**Authors:** M. Angélica Delgado, Omar A. Cáceres, John E. Calderón, Lourdes Balda, Giovanna Sotil, Manuel J. Céspedes

**Affiliations:** ^1^National Reference Laboratory of Bacterial Zoonoses, National Institute of Health, Lima, Peru; ^2^Faculty of Biological Sciences, National University of San Marcos, Lima, Peru; ^3^Biotechnology and Molecular Biology Laboratory, National Institute of Health, Lima, Peru; ^4^Human Medicine Career, Faculty of Health Sciences, Southern Scientific University, Lima, Peru

## Abstract

Leptospirosis is a zoonotic disease caused by the genus *Leptospira,* presenting complex and dynamic epidemiology. To determine the genetic variability and its phylogenetic relationship of *Leptospira* spp isolates from three sources in Iquitos (Peruvian Amazon) from 2002 to 2013, seven MLST genes were analyzed to obtain the *Sequence Type* (ST) and these sequences were concatenated for phylogenetic analysis. The genetic relationship between STs was determined with the goeBURST algorithm and genetic diversity was determined using DnaSP. Of 51 isolates, 48 were pathogenic belonging to five different species: *Leptospira interrogans* Nascimento 2004, *Leptospira santarosai* Feil 2004*, Leptospira noguchii* Haake 2021, *Leptospira borgpetersenii* Levett 2021, and *Leptospira kirschneri* Levett 2021. Of 20 STs identified, 60% corresponded to new genotypes circulating only in Peru. The genotypes ST17, ST37, and ST301 were recorded in rodents and humans. A high intraspecific genetic diversity was demonstrated in *L. noguchi*. The goeBURST analysis revealed three clonal complexes (CCs) and 16 singletons. The STs were found to show high genetic variability and phylogenetic and goeBURST analysis determined that the genotypes found did not form specific groups according to the source of infection or origin, which confirms the zoonotic potential of these STs in an area highly endemic for leptospirosis.

## 1. Introduction

Leptospirosis is a zoonotic disease caused by pathogenic species of *Leptospira* [1,2]. The worldwide annual number of severe cases has been estimated at around one million, and 60,000 deaths worldwide [3]. The highest burden of the disease has been reported in tropical and subtropical countries, where environmental and socioeconomic conditions favor its transmission [1,4]. In Peru, one of the departments that present the highest number of notifications at the national level is Loreto mainly in Iquitos city, located in the Peruvian Amazon region. There, more than 5,618 cases of leptospirosis were reported in 2019 [5] being much higher than in previous years and reflecting the endemicity of the disease in this area. The tropical climate of Iquitos together with other conditions, such as overcrowding in slums and the lack of adequate sanitation, are the main factors that increase the risk of human exposure to the urine of animals infected with *Leptospira* [6, 7].

In humans, leptospirosis has a very wide spectrum of clinical manifestations ranging from mild flu-like symptoms to serious complications such as Weil's disease and Hemorrhagic Pulmonary Syndrome (HPS), with a 40% fatality rate [8]. This disease presents complex and dynamic epidemiology, due to the characteristics of the life cycle of the bacteria, which is involved among humans (susceptible hosts), animals (asymptomatic reservoirs), and ecosystems (environment) [4]. Many domestic and wild animals, including rodents, are the main asymptomatic reservoirs. They play an important role in the cycle of transmission and maintenance of the disease since they carry the bacteria chronically in their renal tubules and they excrete into the environment, from where human acquires the infection [1, 9]. Likewise, the wide distribution of leptospira species in the environment (multiple environmental sources of exposure) reflects their ability and adaptation to survive in different reservoirs and environmental conditions [10, 11]^.^

The *Leptospira* genus is classified in more than 300 serovars based on the structural heterogeneity of the O antigen lipopolysaccharide (LPS) detected by the Cross-agglutinin absorption test (CAAT) [12] and 25 serogroups determined by the microagglutination test (MAT) [1]. Different molecular methods such as DNA-DNA hybridization, 16S rRNA analysis, *Multilocus Sequence Typing* (MLST), and comparative genomics, have been used to identify 22 species of the genus *Leptospira.* Species are classified into three phylogenetic groups: 10 pathogenic, 5 intermediate, and 7 saprophytic, correlated also with the virulence of the bacterium [13]. Currently, with the advent of the relatively inexpensive Whole Genome Sequencing (WGS) and increased interest in metagenomics studies of environmental samples, the number of species has expansion increased, from 22 in 2018 to 64 in 2019 [12].

MLST for the characterization of *Leptospira* variants is a technique based on PCR and followed by sequencing, to assign and characterize alleles present in different target genes and share the information between different laboratories through a database (https://pubmlst.org/organisms/leptospira-spp). Thus, several reports mention the use of MLST for molecular typing in genotypes or *SequenceType* (STs) [14–16]. Likewise, the determination of species in an extremely efficient way through a phylogenetic analysis [10], the genetic diversity, and the characterization of differences in allelic profiles [17]. This last analysis describes the relationships between isolates of a species or population in groups called clonal complexes (CCs) and, in turn, relates CCs to the entire population using goeBURST [18]. CCs are defined as groups of related STs that share at least four loci with at least one member of the group. In general, the founder or ancestral genotype is defined as the ST that presents the highest number of isolates within the same group with variation in a single allele (*single locus variants*, SLV), in two alleles (*double locus variants*, DLV), or three alleles (*triple locus variants*, TLV). Likewise, the STs not assigned to any CC are called singletons, that is, STs differentiated by 4 or more alleles [18].

In Peru, several studies have been carried out for the molecular identification of leptospirosis, thus, the prevalence of *Leptospira* and *Bartonella* species in rodents from the southern Peruvian Amazon has been reported based on 16S metagenomic analysis [19]. Other studies showed the characterization of *Leptospira* from isolates and in biological samples of Iquitos using 16S rRNA gene analysis [6, 20].

Genomic plasticity is known to occur in this genus, thus a genomic island of ∼ 54 kb and a large inversion in chromosome I were reported in the differentiation between the genomes of the Lai and Copenhageni serovars in *L. interrogans* [21]. Also, seven putative genomic islands, ranging in size from 5 to 36kb, were reported in *Leptospira liceraceae* suggesting a history of horizontal gene transfer (HGT) [22]. So, it is necessary to use more robust typing methods, which include several loci with high discriminatory power for different bacterial isolates, easy to apply and standardize, such as MLST [23]. However, the characterization of *Leptospira* spp isolates in Peru is based on the serological test of MAT. Although this method allows typifying serogroups/serovars, it does not discriminate species [1] in addition to being a complicated and laborious technique that requires constant maintenance of reference strains that are used as antigens to obtain the respective antisera [2, 9]. Due to intrinsic differences (genes and antigens) and the existence of serovars that can occur in more than one reservoir and/or host, or that can belong to different species, a small correlation between the molecular and serological classification of *Leptospira* has been detected [24]. It is presumed that the genes that determine the serovar would be related to an HGT of the *rfb* loci, gene cluster associated with the biosynthesis of LPS from the *Leptospira* cell wall [25]. Genetic characterization has greatly contributed to the understanding of the molecular epidemiology of the disease, so both forms of classification are complementary and useful. Accurate identification of disease-causing pathogens is essential for epidemiological surveillance and public health decisions with control and prevention strategies such as the development of effective vaccines [23]. In particular, the identification and genotyping of *Leptospira* plays an important role in understanding the distribution, transmission, and pathogenicity of this disease [15].

In this sense, the present study sought to determine the genetic variability and its phylogenetic relationship of *Leptospira* spp isolates, from different sources and geographic areas of the Peruvian Amazon of Iquitos (a hyperendemic zone for leptospirosis) from 2002 to 2013. Thus, in addition to the MAT test, we evaluated the MLST scheme composed of 7 loci (*housekeeping* genes): *pnt*A (NAD (P) transhydrogenase alpha subunit), *Suc*A (Component 2-oxoglutarate dehydrogenase-decarboxylase), *pfk*B (Ribokinasa), *tpi*A (Triosephosphate isomerase), *mre*A (Protein rodA (Rod Shape-Determining protein rodA), *glm*U (UDP-N-acetylglucosamina pyrophosphorylase) and the *cai*B that encodes Acyl-CoA transferase III/carnitine dehydratase. The new genetic variants identified in this study (and not detected with MAT) were registered in the leptospira database, contributing to the knowledge of new variants of Peruvian leptospires strains throughout the world.

## 2. Materials and Methods

### 2.1. Samples, Strains, and Reactivation of Isolates of *Leptospira* spp

**Figure 1:**
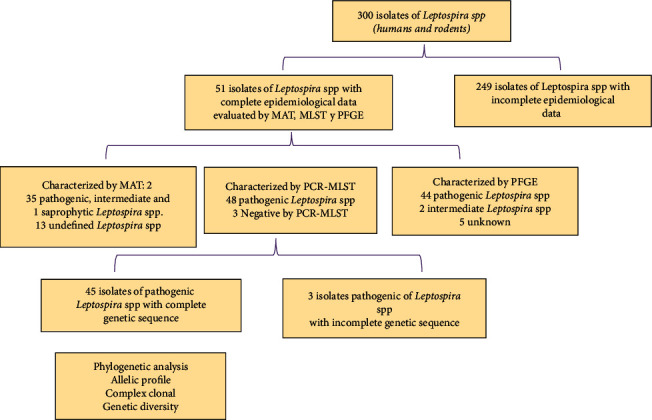
Flowchart of the total samples of *Leptospira* spp isolated from the Iquitos city (Peruvian Amazon), collected from 2002 to 2013. The isolates are stored in the biobank of NRLBZ.

Three hundred (*n* = 300) *Leptospira* isolations were obtained from humans and rodents of different geographical areas from Iquitos city (Peruvian Amazon). The samples were collected between 2002 and 2013 as part of the Peruvian surveillance program for Leptospirosis and as part of a large project called “Dynamic of Leptospirosis Transmission in Maynas-Loreto province 2010–2014,” approved by the Ethics Committee of the National Institute of Health (code: 2-01-05-10-06). *Leptospira* spp isolates (*n* = 51) with their complete epidemiological information and stored in the biobank of the National Reference Laboratory for Bacterial Zoonoses (NRLBZ) (Figure 1).

The reactivation of the isolates was carried out using the liquid medium *Ellinghausen-McCullough-Johnson-Harris* (EMJH), at 28°C, for 6 to 8 weeks. Bacterial growth (up to the log phase) and contamination were observed using a dark field microscope (Eclipse E200, Nikon) at 40X magnification. The isolates with the absence of contamination and a concentration of 1–2 x 10^8^ leptospires/mL (counted in a Petroff Hausser Chamber) were selected to perform the serological and molecular analyzes.

Additionally, six reference strains of pathogenic leptospires (*L. interrogans, L. kirschneri, L. noguchii, L. weilii, L. borgpetersenii,* and *L. santarosai*) provided by the Center for Disease Control and Prevention (CDC-USA) were included for the standardization and evaluation of MLST.

### 2.2. MAT Serological Test

The microscopic agglutination test was performed, where the pure isolates were confronted with a panel of referential antisera from serogroups of 23 serovars indicated in parentheses: Australis (Australis), Autumnalis (Autumnalis), Ballum (Ballum), Bataviae (Bataviae), Canicola (Canicola), Celledoni (Celledoni), Cynopteri, (Cynopteri), Djasiman (Djasiman), Grippotyphosa (Grippotyphosa), Icterohaemorrhagiae (Cophenageni/Mankarso/Icterohaemorrhagiae), Javanica (Javanica), Mini (Georgia) Panama (Panama), Pomona (Pomona), Pyrogenes (Pyrogenes), Sejroe (Wolffi and Hardjo), Shermani (Shermani), Tarassovi (Tarassovi). The serogroups mentioned corresponded to the group of pathogens. The serogroup Iquitos (Varillal) belonged to the intermediate group and Semaranga (Patoc) corresponds to the group of saprophytes. The serogroup was assigned according to the antiserum that produced an agglutination titer ≥800 [2, 9].

### 2.3. Pulse Field Gel Electrophoresis (PFGE) of *Leptospira* spp

The PFGE methodology and analysis were done according to Rivera et al. Reference [26]. Briefly, agarose blocks containing leptospiral DNA were prepared and then digested with 30 U of *NotI* restriction enzyme for 2 hours at 37°C. *Salmonella* serotype Braenderup H9812 was digested with 50 U *XbaI* for use as a standard marker. The agarose blocks containing the digested DNA were placed in the wells of the 1% agarose gel (SeaKem Gold) in 0.5X TBE buffer. The run was carried out using the CHEF MAPPER equipment (Bio-Rad Laboratories) for 18 h at 14°C with recirculating 0.5X TBE buffer and under the following conditions: Initial time of 2.16 s, final time of 35.07 s, an angle of 120° and voltage gradient of 6 V/cm. Gels were stained with ethidium bromide (1 ug/mL) for 20 min and documented with Gel-Doc 2000 (Bio-Rad). The images of the gels were analyzed using the GelCompar II program. Dendrograms were created using UPGMA clustering analysis based on band similarity coefficient with optimization of 1.4% and position tolerance of 1.4%. The database with the PFGE profiles of the 65 reference strains of *Leptospira* spp. was used as a search library for the comparison and identification of serovars of the isolates studied. It was compared with the results obtained by Galloway and Levett for the reference strains [27].

### 2.4. DNA Extraction, Amplification, and Housekeeping Gene Sequencing for MLST

DNA extraction was done using GeneJET Genomic DNA Purification Kit (Thermo Fisher Scientific) for Gram-negative bacteria, following the manufacturer's instructions. The amplification of the internal fragments of seven *housekeeping* genes was performed according to the protocol described by Boonsilp (2013) [15], with slight modifications (Table 1). The PCR was carried out in a total volume of 50 *μ*L of reaction, with final concentrations of 1.5–3.5 mM of MgCl_2_, 0.2 *μ*M–0.6 *μ*M of each primer, 200 *μ*M of dNTP (Applied Biosystems, USA), 1.25 U of Taq DNA polymerase (Invitrogen), and approximately 50 ng of template DNA. The thermal cycling conditions used were, an initial denaturation of 95°C for 2 min, followed by 30 cycles of 95°C for 10 s, 46°C for 15 s and 72°C for 30 s, and a final extension of 72°C for 7 min. The amplified products were evaluated by electrophoresis in 2% agarose gels; products with expected sizes were purified and sequenced for both strands. Sequencing was performed using the same primers from previous PCR, with the BigDye Terminator v3.1 kit (Applied Biosystems), on an ABI 3500XL genetic analyzer (Applied Biosystems). The obtained chromatograms were analyzed using SeqScape v2 program, for editing and exporting the consensus sequence for each allele of the 7 loci.

The sequences of the seven MLST genes obtained from 48 isolates identified as pathogenic leptospires, were concatenated (3111 bp) with the Sequence Matrix v8 program [28]. Additionally, sequences of all alleles available in the MLST schema ^#^1 of Leptospira database (https://pubmlst.org/organisms/leptospira-spp), were obtained, concatenated for each locus, and aligned with those obtained in this study.

### 2.5. MLST Data Analysis

Each allele identified from each sample for each of the seven genes that make up the MLST scheme ^#^1 was assigned a numerical code. Subsequently, the allele combinations of the 7 loci were assigned an allelic profile (known as ST), in the following gene order: *cai*B*-glm*U*-mre*A-*pfk*B*-pnt*A*-sucA-tpi*A. In case of noncoincidence with the database, sequences were verified and sent to the curator of the *Leptospira* MLST database, so new alleles and new STs were assigned, correlative to the existing ones.

### 2.6. Phylogenetic Analysis

For species identification, the concatenated sequences (*n* = 48) were aligned with other 308 concatenated reference sequences obtained from the MLST database, of seven species of the genus *Leptospira* (*L. interrogans*, *L. kirschneri*, *L. noguchi*, *L. kmety, L. borgpeterseni, L. alexanderi, L. weilli,* and *L. santarosai*). The multiple sequence alignment was performed with the Clustal X2 algorithm [29], and the phylogenetic analysis was done using the Maximum Likelihood (ML) method with the Tamura-Nei model with 500 bootstraps implemented in the MEGA *X* program [30].

The phylogenetic relationships of the STs were evaluated considering multiple alignments of the 20 STs sequences identified in the isolates of pathogenic leptospires (with complete allelic profile), and 8 STs of reference strains of *Leptospira* spp. ML analysis was done using the MEGA *X* program with the Tamura-Nei model and 500 bootstraps.

### 2.7. Genetic Diversity

From the concatenated sequences (3111 bp), diversity indices were calculated such as the number of polymorphic sites (S), haplotype diversity (Hd), number of haplotypes (H), and nucleotide diversity (Pi) using the DnaSP program v6 [31], for each population/group of species of the identified Peruvian isolates. It is worth mentioning that considering that the number of polymorphic sites is interpreted based on the number of sequences found and their length; However, the number of sequences is usually highly variable, so the analysis of nucleotide diversity was included (which represents the probability of the sequences that, taken at random, differ in a single site and that does not depend on the number of sequences found).

### 2.8. Assignment of STs in Clonal Complexes

The clustering of STs into clonal complexes (CCs) was done with the goeBURST algorithm, using the PHYLOViZ Online software (https://www.phyloviz.net/goeburst/) [17, 18]. The allelic profiles of 326 STs (up to 12/11/2021) obtained from the Leptospires MLST database (from different countries, sources of isolates, serogroups, and species) were used to determine the CC of samples from this study.

## 3. Results

### 3.1. Typing of Isolates by MAT

Of a total of 51 Peruvian isolates of *Leptospira* spp characterized by MAT, the most predominant serogroup 25.49% (13/51) was Icterohaemorrhagiae with the serovars: Icterohaemorrhagiae/Copenhageni was the most predominant, followed by the serogroup Sejroe (Hardjo/Wolffi) with 11.76% (6/51). Other serogroups were registered but to a lesser extent. Likewise, 25.49% (13/51) of the isolates could not be characterized by MAT, so they were designated as “Not defined” (Supplementary Material, Table S1).

Also, the MAT test allowed the discrimination of two isolates from humans identified as serogroup Iquitos (Varillal), and one from rodents identified as Semaranga (Patoc); belonging to the intermediate and saprophytic groups, respectively (Supplementary Material, Table S1).

### 3.2. Typing of Isolates by PFGE

Of the 51 isolates of *Leptospira* spp characterized by PFGE, four serovars associated with four reference species of *Leptospira* spp were determined with a similarity coefficient ≥78.4%. (1) The species L. *interrogans* (*n* = 28) was made up of four serovars: serovar Icterohaemorrhagiae/Copenhageni (*n* = 22) that also agree with MAT results in 12 samples; the serovar Canicola (*n* = 5) that mostly agree with MAT; one serovar as unknown (*n* = 1) but identified as serogroup Bataviae by MAT. (2) The species L. *santarosai* (*n* = 14) is associated with serovars that could not be defined by PFGE and different serogroups by MAT. (3) The species L. *noguchii* (*n* = 2) was associated with the serovar *Proechimys.* (4) The species L. *licerasiae* (*n* = 2) was associated with the serovar Varillal (Supplementary material, Table S2).

### 3.3. Typing of Isolates by MLST

#### 3.3.1. Species Identification

**Figure 2:**
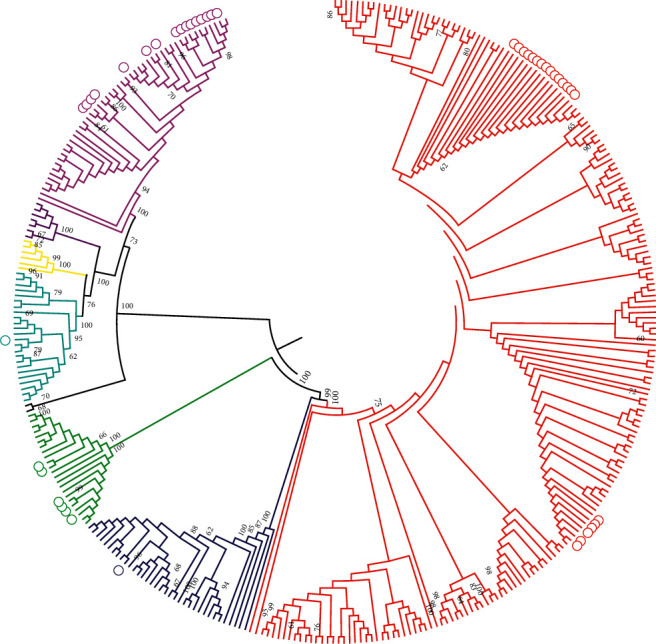
Maximum Likelihood (ML) analysis using the Tamura-Nei model of concatenated sequences of 7 MLST genes for the determination of pathogenic *Leptospira* spp species. Circles indicate the sequences (n=48) obtained in this study. The colors indicate the different reference sequences (n=308) of the genus *Leptospira* analyzed (red *L. interrogans*, blue *L. kirschneri*, green *L. noguchi*, black *L. kmety*, light blue *L. borgpetersenii*, yellow *L. alexanderi*, purple *L. weilli*, and pink *L. santarosai*). The numbers indicate the bootstrap value.

Of 51 Peruvian isolates of *Leptospira* spp, 48 were identified as pathogenic leptospires and 3 as PCR-MLST negative (nonpathogenic), therefore they were not considered for this study. In addition, the ML tree showed that isolates from different distribution areas were discriminated in 5 clades, with high bootstrap values (100%). The isolates were identified as: *L. interrogans* (*n* = 21), *L. santarosai* (*n* = 17*), L.noguchii* (*n* = 8), *L. borgpetersenii* (*n* = 1) and *L. kirschneri* (*n* = 1) (Figure 2).

#### 3.3.2. Obtaining STs by MLST

The MLST analysis of the pathogenic leptospires isolates, discriminated 88 alleles, of which 75 were known and 13 alleles not previously registered (new). We registered between 10 and 16 alleles per locus. 13 alleles were identified for the *glm*U gene (with one new allele), 14 alleles for the *pnt*A gene (three new), 13 alleles for the *suc*A gene (two new), 11 for the *tpi*A gene (two new), 16 for the *pfk*B gene (three new), 11 for the *mre*A gene (two new) and 10 alleles for the *cai*B gene (one new allele). The distribution of each sample by locus is shown (Supplementary material, Figure S1). On the other hand, 20 STs were registered, of which 12 STs were new and reported only in Peru (Table 2).

Of the 48 isolates analyzed, three of them (LEP_150, LEP_151, and LEP_171) were not possible to determine the loci sequences (*pfk*B, *pnt*A, and *cai*B, respectively), therefore for these isolates it was not possible to define ST due to their incomplete allelic profile, but its identification was carried out up to the species level. These sequences were also excluded from the analysis for the identification of CCs and genetic diversity.

#### 3.3.3. Genetic Diversity

High intraspecific genetic diversity was observed in *L. noguchii* (Hd = 0.933 ± 0.122) and *L. santarosai* (Hd = 0.908 ± 0.063). Likewise, *L. santarosai* presented slightly more polymorphic sites (4.4%) compared to *L. noguchii* (4.27%) (Table 3).

**Figure 3:**
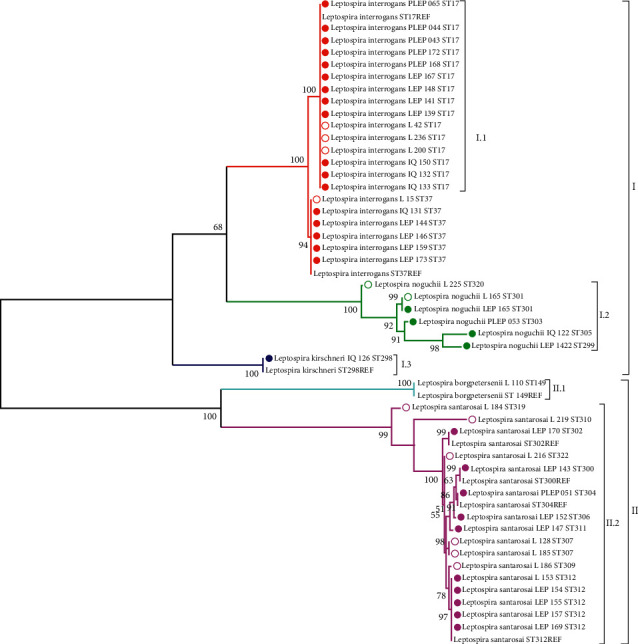
Phylogenetic relationships of the 20 STs were obtained from 45 isolates and 8 STs of reference strains of *Leptospira* spp based on the Maximum Likelihood (ML) method. The colored circles correspond to human isolates and uncolored to animal samples (red, *L. interrogans*; green, *L. noguchii*; blue, *L. kirschneri*; light blue *L. borgpetersenii*; and pink, *L. santarosai*).

The phylogenetic relationships between the 20 genotypes (STs) of the 45 pathogenic leptospires species found in the present study, showed differentiation into two groups, which partially coincided with the identified serogroups. Group I was formed by three subgroups integrated by *L. interrogans* (I.1)*, L. noguchii* (I.2), and *L. kirschneri* (I.3); while group II consisted of two subgroups formed by the species *L. borgpetersenii* (II.1), and *L. santarosai* (II.2) (Figure 3).

#### 3.3.4. Identification of Clonal Complexes

**Figure 4:**
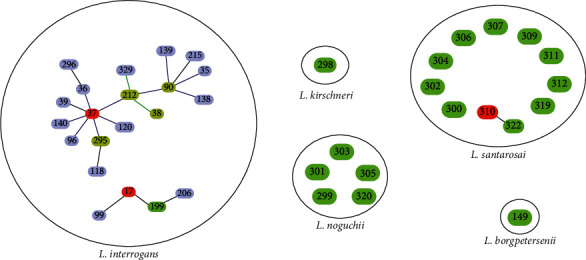
Graphical representation of the association between STs of Peruvian isolates together with those of the MLST database of L. species by goeBURST analysis. Three CCs (red) and 16 singletons (green) found in the present study were observed. The CCs were constructed from connections between STs allowing up to 3 allelic variants (TLVs). The 16 singletons were present in *L. kirschneri*, *L. noguchii, L. santarrosai,* and *L. borgpetersenii* species.

The goeBURST algorithm of the Peruvian 20 STs in conjunction with those of the *Leptospira* MLST database (312 STs) allowed for establishing 3 clonal complexes: CC17, CC37, and CC310 and 16 singletons (Figure 4).

Within the clonal complexes CC17 and CC37, both *L. interrogans,* ST17, and ST37 were designated as the most frequent and founder clones of each CC. The CC149 belongs to *L. borgpetersenii* and consisted of two genotypes: ST149 (known) and ST321 (new). The CC310 of *L. santarosai* was composed of genotypes ST310 and ST322, both STs determined only for Peru, and each one represented by a *Leptospira* isolates. The CC310 complex is SLV-type CCs that are linked only by two genotypes (Figure 4).

Genetic variability was also represented in the 16 singletons found in this study. Of these, 10 singletons (ST299, ST301, ST303, ST305, ST306, ST307, ST309, ST311, ST319, ST320) were identified as “new” and circulating only in the Peruvian Amazon, while the other 6 known singletons (ST149, ST298, ST300, ST302, ST304, ST312) have also been reported in other countries according to the MLST database (Table 2).

## 4. Discussion

New genetic variants of *Leptospira* spp were detected in this study, by MLST, circulating in the Peruvian Amazon. This is the first MLST molecular typing study (based on 7 housekeeping genes) carried out in Peru, from isolates of pathogenic leptospires from different sources and geographic areas of Iquitos city, collected over 11 years (2002–2013). Iquitos, located in the Peruvian Amazon, is considered a hyperendemic zone for leptospirosis [6, 20]. Local epidemiological studies (associated with isolates recovered from an outbreak) and global (to know how the strains that cause diseases in a geographic area with isolates worldwide) are relevant to contribute to the application of prevention and control strategies for the leptospirosis transmission.

The 51 isolates of *Leptospira* spp were also evaluated by PFGE. Of these, only 37 results are concordant between both methods (Table S2). Although PFGE is the gold standard for molecular subtyping of *Leptospira* [27]; this technique does not have sufficient discriminating power to determine all the serovars of the bacteria. A possible explanation is the lack of reference strains that include all the existing *Leptospira* serovars and that only allowed us to identify the most common serovars such as Icterohaemorrhagiae/Copenhageni and Canicola of the species *L. interrogans*, which were the most predominant and coincided with MAT and MLST results. This situation was not observed when dealing with relatively new serovars and species such as *L. noguchii*, *L. borgpetersenii,* and *L. santarosai*.

Considering the high genetic diversity of *Leptospira* spp at the serovar level in Peruvian isolates and specifically in the Peruvian Amazon [26], in addition to what was previously described, it was necessary to apply more precise molecular methods such as MLST, which allowed us to characterize more accurately the pathogenic species as well as the genotypes of the bacteria.

Of the five species identified (by ML), *L. interrogans* was the most predominant, with 43.75% (21/48) of the total analyzed, and concordant with two serogroups: Icterohaemorrhagiae and canicola defined by MAT and PFGE. Several reports mention that *L. interrogans* is widely distributed in the world and is associated with several outbreaks of leptospirosis in animals, including humans. In China, this species has been the most predominant for 50 years, with 90.83% (109/120) [32]. Likewise, 76% of the cases that occurred in an outbreak in Thailand during 2007 were recorded to correspond to *L. interrogans* serovar Autumnalis and ST34 [16].

In general, a high genetic diversity (Hd = 0.867 ± 0.041) was registered in the total of isolates identified in this study, showing the highest intraspecific genetic diversity in *L. noguchii* (Hd = 0.933 ± 0.122) followed by *L. santarosai* (Hd = 0.908 ± 0.063) (Table 3). Similar results were found in MLST studies carried out in cattle in Brazil, where a great genetic diversity (*H* = 0.96 ± 0.223) was observed for *L. noguchii* [33]. Likewise, another study on domestic and wild reservoir animals mentioned *L. santarosai* as the most interesting species with high intraspecific diversity (Hd = 0.942 ± 0.034) [34]. These two species, in our case, only limited their presence in humans and rodents, which could lead to differences in virulence, antigenicity, and adaptability of these strains to their hosts [10, 33]. However, there is the possibility of finding these species in other different reservoirs, such as domestic animals, and their circulation in different ecosystems, confirming their zoonotic potential.

Is important to mention that a high percentage (60%, 12/20) of registered genotypes (STs) were considered “new,” apparently circulating only in Peru. It should be noted that the STs were found in different sources of isolation; and that 12 of the new STs were derived from new alleles at various loci, while the new ST309 was generated by a different combination of alleles already known and present in various STs (300, 302, 304, 306, 307, 309, 310, 311, 312, 319, 322). This evidence highlights the potential of the MLST to explore the transmission and circulation of genotypes between reservoirs and humans, both during outbreaks and in epidemiological studies [15, 32].

Some genotypes found in this study have crossed the barrier between species, evidenced by the presence of genotypes ST17, ST37, and ST301 (the latter “new”) in humans and rodents, which reaffirms the fact that reservoirs of the genus *Rattus* spp are an important source of transmission of leptospirosis. The genotype ST17 is known to be virulent to its hosts and is generally part of a zoonotic transmission cycle, involving humans, rats, and dogs [15, 16, 32]. On the other hand, the genotype ST37 has also been reported in Argentina, Brazil [35, 36], and Thailand [15], as responsible for leptospirosis in humans. In addition, ST17 and ST37 were recorded in this study within the *L. interrogans* species, forming two different clusters with high statistical support, being the most frequent and closely related, since they share a recent common ancestor (Figure 3).

Also, something to highlight is that all the genotypes (ST299, ST305, ST303, ST301, and ST320) identified in the isolates of *L. noguchii* were registered as news and found so far only in Peru. In addition, a close phylogenetic relationship was not observed between its members, being ST320 the most ancestral and all of them grouped in a cluster with high statistical support. The presence of ST301 in two isolates, one from humans (collected in 2003) and the other from rodents (from 2013), would reflect the occurrence of the circulation of the same genotype over time and in different sources. Similarly, other genotypes have been described with the capacity to infect a wide variety of domestic animal hosts, as well as rats and bats, at the same time. Also, serious clinical cases have been reported in humans in Brazil [33].

The genotype ST298, characterized in the *L. kirshneri* species from a 2012 human sample, could not be characterized by MAT and PFGE (Supplementary material, Table S2). According to the MLST database, this genotype is restricted to a small number of isolates, one of swine from the United States and 3 isolates of unknown origin and source (https://pubmlst.org/organisms/leptospira-spp). However, there are other different genotypes reported within *L. kirschneri* in different geographical areas, such as the genotype ST117 isolated from domestic animals (cattle and horses), ST100 isolated from rodents [37], ST110 from horse, and ST124 isolated from capibara [34]. All these genotypes were implicated in the transmission of leptospirosis and their zoonotic implications.

It is known that many genes of the *L. interrogans* genome are related to the high rate of transmission through water, which does not occur with *L. borgpetersenii* due to a genetic decay process restricted to survival within the host, decreasing its transmissibility [38]. In our study, the ST149 genotype was recorded as *L. borgpetersenii* by MLST, but as *L interrogans* by PFGE, in one rodent isolate (L_110) (Supplementary material, Table S2). It should be noted that this ST149 is widely distributed in Asia [15] and less frequently in European countries, such as Portugal where this species was isolated from rodents [37]. Likewise, in Sardinia-Italy, 9 out of 23 isolates from various wild animals (rodents, hedgehogs, and foxes) corresponded to this genotype, involved in the natural cycle of leptospirosis transmission [39]. On the other hand, there are other genotypes within this same species, such as the ST145 of serovar Javanica, isolated mostly from rodents, implicated as an important source of transmission of human leptospirosis in India [11].

On the other hand, most of the genotypes identified in isolates of the species *L. santarosai* (eleven new STs and four knowns) could not be characterized by serology, so they were determined as “not defined” by MAT (Supplementary Material, Table S2) and “unknown” by PFGE. According to ML analyzes, these genotypes were not closely related. Thus, the genotypes ST300, ST304, ST306, and ST311 clustered in a subgroup composed only of human isolations. A second subgroup was formed by ST307 of two isolations from rodents (from 2005 to 2012), evidencing its circulation over time. Another third subgroup was integrated by two genotypes, ST309 (from rodents) and ST312; this last one showed a well-defined cluster with a high level of confidence made up of 5 human isolates that remained circumscribed between 2004 and 2005, not being found in the following years. Finally, the genotypes ST322, ST302, ST310, and ST319 would become the most ancestral, determined in the phylogenetic tree with good statistical support (Figure 2). Other genotypes of this species different from those found in our study, have been reported as causing serious illness and death in humans in Sri Lanka [15]. Likewise, a great diversity of “new” genotypes in cattle have been reported in Brazil [34]. Due to their importance as infectious agents and even more so because of the presence of several genotypes reported in this work, it is necessary to study them in more detail and with a greater number of samples, in different reservoirs.

The characterization of leptospires using MLST scheme ^#^1, based on allelic profiles, allowed the identification of three CCs of leptospires, grouped independently of their source of infection and geographic area. That is, there were no isolates from certain epidemiological origins that were grouped into specific genetic lines, without an association between STs with sources of the origin or geographical origin. On the contrary, a clustering of strains of human and animal origin was evidenced, in the same ST and/or CC, as was the case of the ST17, ST37, and ST301 genotypes (Table 2). This grouping also corresponded with the observed ML. The two main CCs : CC17 (15 isolates) and CC37 (6 isolates) corresponded to *L. interrogans* and presented as the ancestor and more frequent clone ST17 and ST37, respectively; and were related to other STs of the global leptospira database through of SLVs or DLVs (Figure 4). The goeBURST results showed that the species are confined within different CCs, there being no coexistence of isolates of different species in the same CC. This result provides robustness to the MLST evaluation. On the other hand, despite the association of ST310 and ST322 evidenced in the goeBURST analysis as CC310, both genotypes showed a different and distant evolutionary diversification in the phylogenetic tree with many nucleotide differences (Figure 3). The STs that makeup CC310 (ST310 and ST322) result from an allelic variant at loci *mre*A and the combination of alleles already known, that is, ST310 presents allele 2 at the *suc*A loci, which is also found in other STs (ST17 and ST322). ST322 also presents allele 47 at the *suc*A loci that were also found in other STs (ST304, ST306, ST307, ST309, ST311, and ST322) (Table 2).

These findings could be associated with previously described situations, such as (1) the appearance of a high number of polymorphisms in a gene that could be considered evidence of the existence of recombination in a bacteria population [40]; (2) the evidence of statistically significant putative recombination found between the *suc*A pathogenic and *pfk*B genes, observed in isolates of pathogenic leptospires in Argentina, variants that could generate new alleles and therefore new STs, as is the case of the new STN1(35); (3) evidence of HGT in *Leptospira* generating the appearance of an allelic profile that seems to arise from the combination between two other STs, as happened in the case of STN2 found in an MLST study for *Leptospira* in Argentina. Its profile was not made up of new alleles, but consisted of a new combination of alleles already known present in ST58 (*glm*U, *pnt*A, *suc*A, *fad*D, and *pnt*A) and ST17 (*pfk*B and *mre* A) [35]; (4) genetic variations in two *suc*A and *pfk*B loci in six isolates of leptospires described in an MLST study in India, where they classified it as distantly related (DR) strains, assuming that they could be related to the supposed HGT that can occur between leptospires species [11]. On the other hand, there are other studies of the MLST scheme ^#^ 2 for *Leptospira* in which it contains the genes of scheme ^#^ 1 (*glm*U, *mre*A, and *pnt*A) and which have shown high levels of genetic recombination and HGT for strains of the genus *Leptospira* [36].

A limitation of the study was associated with the laboriousness and its slight complexity in the processing of the MLST method, in addition to requiring contamination-free isolates of leptospires and a good microbial concentration. However, there is also the possibility of optimizing the MLST directly from clinical samples or having other technologies. Thus, for example, the application of NGS technologies would allow us to expand the genetic variability studies of Leptospira more quickly and efficiently, as well as offer advantages when working with a larger number of samples from different isolation sources and geographical areas.

## 5. Conclusions

The identification of new genotypes given in this study, together with all the epidemiological information, has contributed to the increase of records in MLST database of *Leptospira,* being the first work of its type carried out in Peru. The determination of genotypes of *Leptospira* spp by MLST, of rodents as a source of transmission of leptospirosis in a hyperendemic area and its association with severe clinical cases, is of great relevance and utility for the molecular epidemiology of this pathogen. Indeed, these contributions will make it possible to suggest adequate measures regarding the rodent control strategy for reducing the transmission of the disease from animals to humans.

## Figures and Tables

**Table 1:** List of MLST loci, primers, and amplification conditions used for the typing of pathogenic Leptospira spp, based on the method described by Boonsilp et al., 2013 with modifications.

Loci	Primers	Nucleotide sequence (5´ a 3´)	MgCl2 (mM)	Primers concentration (*μ*M)	PCR size (pb)	loci size (pb)	Localization in chromosome I
pntA	pntA-FM	TAG GAA ARA TGA AAC CRG GAA C	3.5	0.2	621	525	56347–56871
	pntA-RM	AAG AAG CAA GAT CCA CAA YTA C					

caiB	caiB-F	CAACTTGCGGAYATAGGAGGAG	3.5	0.2	650	402	1562845–1563246
	caiB-R	ATTATGTTCCCCGTGAYTCG					

glmU	glmU-FM	AGG ATA AGG TCG CTG TGG TA	3.5	0.2	650	444	3784955–3784512
	glmU-RM	AGT TTT TTT CCG GAG TTT CT					

tpiA	pntA-FM	TTG CAG GAA ACT GGA AAA TGA AT	3.5	0.2	639	426	1694673–1694248
	pntA-RM	GTTTTACRGAACCHCCGTAGAGAAT					

pfkB	pfk B-FM	CGGAGAGTTTTATAARAAGGACAT	1.5	0.2	588	432	1386553–1386984
	pfk B-RM	AGAACACCCGCCGCAAAACAAT					

sucA	sucA-FM	TCA TTC CAC TTY TAG ATA CGA T	3.5	0.6	640	447	1227474–1227920
	sucA-RM	TCTTTTTTGAATTTTTGACG					

mreA	mreA-FM	GGC TCG CTC TYG ACG GAA A	3.5	0.6	719	435	2734550–2734116
	mreA-RM	TCCRTAACTCATAAAMGACAAAGG					

**Table 2:** Species, allelic profiles, and STs know and news of pathogenic Leptospira spp, identified from human and rodent isolates from the Iquitos city (Peruvian Amazon), collected from 2002 to 2013.

N°	Code of sample	Sources of isolates	Places of isolates	Date of isolates	Species by PFGE	Species by MLST	glmU	pntA	sucA	tpiA	pfkB	mreA	caiB	ST
1	IQ_131	Human	San Juan Bautista	26/04/2013	*L. interrogans*	*L. interrogans*	3	3	3	3	4	5	5	37
2	IQ_132	Human	Belén	7/05/2013	*L. interrogans*	*L. interrogans*	1	1	2	2	10	4	8	17
3	IQ_133	Human	Belén	14/05/2013	*L. interrogans*	*L. interrogans*	1	1	2	2	10	4	8	17
4	IQ_150	Human	Belén	3/06/2013	*L. interrogans*	*L. interrogans*	1	1	2	2	10	4	8	17
5	LEP_139	Human	Iquitos	28/05/2003	*L. interrogans*	*L. interrogans*	1	1	2	2	10	4	8	17
6	LEP_141	Human	Iquitos	11/06/2003	*L. interrogans*	*L. interrogans*	1	1	2	2	10	4	8	17
7	LEP_144	Human	Iquitos	3/10/2003	*L. interrogans*	*L. interrogans*	3	3	3	3	4	5	5	37
8	LEP_146	Human	Iquitos	14/11/2003	*L. interrogans*	*L. interrogans*	3	3	3	3	4	5	5	37
9	LEP_148	Human	Iquitos	1/09/2004	*L. interrogans*	*L. interrogans*	1	1	2	2	10	4	8	17
10	LEP_159	Human	San Juan Bautista	9/08/2004	*L. interrogans*	*L. interrogans*	3	3	3	3	4	5	5	37
11	LEP_167	Human	San Juan Bautista	2/07/2004	*L. interrogans*	*L. interrogans*	1	1	2	2	10	4	8	17
12	LEP_168	Human	San Juan Bautista	28/05/2004	*L. interrogans*	*L. interrogans*	1	1	2	2	10	4	8	17
13	LEP_172	Human	Belén	3/06/2003	*L. interrogans*	*L. interrogans*	1	1	2	2	10	4	8	17
14	LEP_173	Human	Belén	25/08/2003	*L. interrogans*	*L. interrogans*	3	3	3	3	4	5	5	37
15	PLEP043	Human	Belén	17/02/2012	*L. interrogans*	*L. interrogans*	1	1	2	2	10	4	8	17
16	PLEP044	Human	Belén	17/02/2012	*L. interrogans*	*L. interrogans*	1	1	2	2	10	4	8	17
17	IQ_126	Human	Belén	9/08/2012	*Unknown*	*L. kirschneri*	78	20	13	22	33	18	23	298
18	LEP_142	Human	Iquitos	22/08/2003	*L. interrogans*	*L. noguchi*	81a	82	88a	79a	114a	78a	72a	299b
19	LEP_143	Human	Iquitos	14/10/2003	*L. santarosai*	*L. santarosai*	40	55	85	50	103	47	43	300
20	LEP_165	Human	San Juan Bautista	10/11/2003	*L. noguchii*	*L. noguchi*	38	89a	45	79a	113a	40	40	301b
21	LEP_170	Human	Iquitos	7/12/2004	*L. santarosai*	*L. santarosai*	80	90	54	50	74	47	71	302
22	PLEP053	Human	Belén	11/05/2012	*L. noguchii*	*L. noguchi*	38	91a	46	46	46	40	40	303b
23	PLEP065	Human	Belén	20/08/2012	*L. interrogans*	*L. interrogans*	1	1	2	2	10	4	8	17
24	PLEP051	Human	Belén	31/05/2012	*L. santarosai*	*L. santarosai*	45	51	47	50	103	47	43	304
25	IQ_122	Human	Belén	14/09/2012	*Unknown*	*L. noguchi*	35	92	86	39	114a	79	34	305b
26	LEP_152	Human	San Juan Bautista	4/09/2003	*L. santarosai*	*L. santarosai*	40	88a	47	50	108	47	43	306b
27	LEP_147	Human	Iquitos	7/02/2004	*L. santarosai*	*L. santarosai*	73	90	47	50	108	81a	43	311b
28	LEP_153	Human	San Juan Bautista	27/10/2004	*L. santarosai*	*L. santarosai*	40	53	87	80	115	80	43	312
29	LEP_154	Human	San Juan Bautista	9/12/2004	*L. santarosai*	*L. santarosai*	40	53	87	80	115	80	43	312
30	LEP_155	Human	San Juan Bautista	21/01/2005	*L. santarosai*	*L. santarosai*	40	53	87	80	115	80	43	312
31	LEP_157	Human	San Juan Bautista	12/07/2004	*Unknown*	*L. santarosai*	40	53	87	80	115	80	43	312
32	LEP_169	Human	Iquitos	17/12/2004	*L. santarosai*	*L. santarosai*	40	53	87	80	115	80	43	312
33	LEP_150	Human	Iquitos	12/04/2005	*L. santarosai*	*L. santarosai*	79	90	82	50	74∼	47	43	x
34	LEP_151	Human	San Juan Bautista	4/09/2003	*Unknown*	*L. noguchi*	38	89∼	46	46	117	40	36	x
35	LEP_171	Human	Iquitos	28/04/2004	*Unknown*	*L. noguchi*	35	92	86	39	112	79	62∼	x
36	L_236	Rodent	Punchana	17/10/2013	*L. interrogans*	*L. interrogans*	1	1	2	2	10	4	8	17
37	L_42	Rodent	Belén	15/12/2011	*L. interrogans*	*L. interrogans*	1	1	2	2	10	4	8	17
38	L_110	Rodent	Punchana	10/01/2013	*L. interrogans*	*L. borgpetersenii*	24	32	30	36	67	26	12	149
39	L_128	Rodent	Iquitos	3/10/2012	*L. interrogans*	*L. santarosai*	82	53	47	82a	55	81a	43	307b
40	L_185	Rodent	San Juan Bautista	6/06/2005	*L. santarosai*	*L. santarosai*	82	53	47	82a	55	81a	43	307b
41	L_186	Rodent	Punchana	1/10/2004	*L. interrogans*	*L. santarosai*	40	53	47	50	116	80	43	309b
42	L_219	Rodent	Iquitos	13/10/2004	*L. santarosai*	*L. santarosai*	80	53	2	50	74	81a	43	310b
43	L_165	Rodent	Punchana	27/08/2013	*L. interrogans*	*L. noguchi*	38	89a	45	79a	113a	40	40	301b
44	L_15	Rodent	Belén	17/08/2011	*L. interrogans*	*L. interrogans*	3	3	3	3	4	5	5	37
45	L_200	Rodent	Punchana	1/10/2004	*L. interrogans*	*L. interrogans*	1	1	2	2	10	4	8	17
46	L_216	Rodent	San Juan Bautista	30/10/2004	*L. santarosai*	*L. santarosai*	80	53	47	50	74	81a	43	322b
47	L_184	Rodent	San Juan Bautista	6/06/2005	*L. santarosai*	*L. santarosai*	79	55	89a	50	108	58	43	319b
48	L_225	Rodent	Belén	20/09/2013	*L. interrogans*	*L. noguchi*	38	46	45	41	117a	5	36	320b

New alleles (a) and new STs (b) are in red and undefined alleles are indicated with (∼). Samples in bold indicates the three isolates with incomplete allelic profiles.

**Table 3:** Genetic diversity parameters of isolates of pathogenic leptospires from the Iquitos (Peruvian Amazon), collected from 2002 to 2013.

Genetic diversity parameters	Species of pathogenic leptospires					All species
	*L. interrogans*	*L. santarosai*	*L. noguchii*	*L. borpetersenii*	*L. kirschneri*	
Numbers of sequences	21	16	6	1	1	46
Numbers of haplotypes	2	11	5	1	0	20
Genetic diversity (SD)	0.429 ± 0.089	0.908 ± 0.063	0.933 ± 0.122	0	0	0.867 ± 0.041
Number of polymorphic sites (%)	16 (0.51%)	137 (4.4%)	133 (4.27%)	0	0	900(28.92%)
Nucleotide diversity	0.00220	0.01082	0.01834	0	0	0.10736

## Data Availability

The MLST data used to support the findings of this study are available from Dr. Angelica Delgado (angelicadb.2741@gmail.com) upon request.

## Supplementary Materials

Supplementary Materials
*Figure S1.* ML phylogenetic trees of each locus of MLST were analyzed by the MEGA X program: glmU, pntA, sucA, tpiA, pfkB, mreA y sucA. The trees show the distributions of 45 isolates of pathogenic leptospires. Red : *L. interrogans*; yellow: *L. kirschneri*; green: *L. nogouchii*; light blue: *L. borgpetersenii*; and pink: *L. santarosai. Table S1.* Serogroups and serovars were identified by MAT in isolates of Leptospira spp. from humans and rodents from Iquitos (Peruvian Amazon), collected from 2002 to 2013. *Table S2*. Results of MAT, MLST, and PFGE were obtained from 51 isolates of *Leptospira* spp. from the Iquitos city (Peruvian Amazon), collected from 2002 to 2013.
