# Supplementary material for: New Genetic Variants of Leptospira spp Characterized by MLST from Peruvian Isolates
Source: J Trop Med. 2022 Sep 22;2022:4184326. doi: 10.1155/2022/4184326 (PMC9553527; doi:10.1155/2022/4184326)
Supplement: Supplementary Materials — Figure S1. ML phylogenetic trees of each locus of MLST were analyzed by the MEGA X program: glmU, pntA, sucA, tpiA, pfkB, mreA y sucA. The trees show the distributions of 45 isolates of pathogenic leptospires. Red : L. interrogans; yellow: L. kirschneri; green: L. nogouchii; light blue: L. borgpetersenii; and pink: L. santarosai. Table S1. Serogroups and serovars were identified by MAT in isolates of Leptospira spp. from humans and rodents from Iquitos (Peruvian Amazon), collected from 2002 to 2013. Table S2. Results of MAT, MLST, and PFGE were obtained from 51 isolates of Leptospira spp. from the Iquitos city (Peruvian Amazon), collected from 2002 to 2013. [file 4184326.f1.zip › Table S2.docx]

**Table S2.** Results of MAT, MLST, and PFGE were obtained from 51 isolates of *Leptospira* spp from the Iquitos city (Peruvian Amazon), collected from 2002 to 2013.

| **N°** | **Serogrupo/serovar by MAT** | **Species by MLST** | **Species by PFGE** | **Serovar by PFGE** |
| --- | --- | --- | --- | --- |
| 1 | Undefined | *L. interrogans* | *L. interrogans* | Canicola |
| 2 | Icterohaemorrhagiae (6400) | *L. interrogans* | *L. interrogans* | Icterohaemorrhagiae/Copenhageni |
| 3 | Icterohaemorrhagiae (6400) | *L. interrogans* | *L. interrogans* | Icterohaemorrhagiae/Copenhageni |
| 4 | Icterohaemorrhagiae (6400) | *L. interrogans* | *L. interrogans* | Icterohaemorrhagiae/Copenhageni |
| 5 | Icterohaemorrhagiae (6400) | *L. interrogans* | *L. interrogans* | Icterohaemorrhagiae/Copenhageni |
| 6 | Icterohaemorrhagiae (3200) | *L. interrogans* | *L. interrogans* | Icterohaemorrhagiae/Copenhageni |
| 7 | Canicola (6400) | *L. interrogans* | *L. interrogans* | Canicola |
| 8 | Canicola (6400) | *L. interrogans* | *L. interrogans* | Canicola |
| 9 | Icterohaemorrhagiae (12800) | *L. interrogans* | *L. interrogans* | Icterohaemorrhagiae/Copenhageni |
| 10 | Canicola (12800) | *L. interrogans* | *L. interrogans* | Canicola |
| 11 | Icterohaemorrhagiae (12800) | *L. interrogans* | *L. interrogans* | Icterohaemorrhagiae/Copenhageni |
| 12 | Icterohaemorrhagiae (12800) | *L. interrogans* | *L. interrogans* | Icterohaemorrhagiae/Copenhageni |
| 13 | Icterohaemorrhagiae (12800) | *L. interrogans* | *L. interrogans* | Icterohaemorrhagiae/Copenhageni |
| 14 | Canicola (12800) | *L. interrogans* | *L. interrogans* | Canicola |
| 15 | Undefined | *L. interrogans* | *L. interrogans* | Icterohaemorrhagiae/Copenhageni |
| 16 | Icterohaemorrhagiae (12800) | *L. interrogans* | *L. interrogans* | Icterohaemorrhagiae/Copenhageni |
| 17 | Undefined | *L. kirschneri* | Unrealized | Unknown |
| 18 | **Bataviae(12800)** | ***L. noguchii*** | ***L. interrogans*** | **Unknown** |
| 19 | Undefined | *L. santarosai* | *L. santarosai* | Unknown |
| 20 | Bataviae (12800) | *L. noguchii* | *L. noguchii* | Proechimys |
| 21 | Pyrogenes (12800) | *L. santarosai* | *L. santarosai* | Unknown |
| 22 | Undefined | *L. noguchii* | *L. noguchii* | Proechimys |
| 23 | Icterohaemorrhagiae (12800) | *L. interrogans* | *L. interrogans* | Icterohaemorrhagiae/Copenhageni |
| 24 | Undefined | *L. santarosai* | *L. santarosai* | Unknown |
| 25 | **Undefined** | ***L. noguchii*** | ***Unknown*** | **Unknown** |
| 26 | Icterohaemorrhagiae(800) | *L. santarosai* | *L. santarosai* | Unknown |
| 27 | Tarassovi (200) | *L. santarosai* | *L. santarosai* | Unknown |
| 28 | Sejroe (1600) | *L. santarosai* | *L. santarosai* | Unknown |
| 29 | Sejroe (1600) | *L. santarosai* | *L. santarosai* | Unknown |
| 30 | Sejroe (800) | *L. santarosai* | *L. santarosai* | Unknown |
| 31 | Sejroe (800) | *L. santarosai* | *Unrealized* | Unknown |
| 32 | Sejroe (1600) | *L. santarosai* | *L. santarosai* | Unknown |
| 33 | Tarassovi (200) | *L. santarosai* | *L. santarosai* | Unknown |
| 34 | Bataviae (25600) | *L. noguchii* | *Unrealized* | Unknown |
| 35 | **Australis(25600)** | ***L. noguchii*** | ***Unknown*** | **Unknown** |
| 36 | Undefined | *L. interrogans* | *L. interrogans* | Icterohaemorrhagiae/Copenhageni |
| 37 | Undefined | *L. interrogans* | *L. interrogans* | Icterohaemorrhagiae/Copenhageni |
| 38 | **Ballum(800)** | ***L. borgpetersenii*** | ***L. interrogans*** | **Icterohaemorrhagiae/Copenhageni** |
| 39 | **Undefined** | ***L. santarosai*** | ***L. interrogans*** | **Icterohaemorrhagiae/Copenhageni** |
| 40 | Georgia (800) | *L. santarosai* | *L. santarosai* | Unknown |
| 41 | **Undefined** | ***L. santarosai*** | ***L. interrogans*** | **Icterohaemorrhagiae/Copenhageni** |
| 42 | Undefined | *L. santarosai* | *L. santarosai* | Unknown |
| 43 | **Bataviae (800)** | ***L. noguchii*** | ***L. interrogans*** | **Icterohaemorrhagiae/Copenhageni** |
| 44 | Canicola (6400) | *L. interrogans* | *L. interrogans* | Icterohaemorrhagiae/Copenhageni |
| 45 | Icterohaemorrhagiae (12800) | *L. interrogans* | *L. interrogans* | Icterohaemorrhagiae/Copenhageni |
| 46 | Shermani (25600) | *L. santarosai* | *L. santarosai* | Unknown |
| 47 | Sejroe (800) | *L. santarosai* | *L. santarosai* | Unknown |
| 48 | **Undefined** | ***L. noguchii*** | ***L. interrogans*** | **Icterohaemorrhagiae/Copenhageni** |
| 49 | Varillal (6400) | Negative PCR-MLST | *L. licerasiae* | Varillal |
| 50 | Varillal (3200) | Negative PCR-MLST | *L. licerasiae* | Varillal |
| 51 | Patoc (800) | Negative PCR-MLST | *L. interrogans* | Icterohaemorrhagiae/Copenhageni |

Letters in bold show discordance, at the species level, between MLST and PFGE
